# Empowering Girls and Women through Hookworm Prevention

**DOI:** 10.4269/ajtmh.17-0934

**Published:** 2018-04-02

**Authors:** Peter J. Hotez

**Affiliations:** Texas Children’s Hospital Center for Vaccine Development, Department of Pediatrics and Molecular Virology and Microbiology, National School of Tropical Medicine, Baylor College of Medicine, Houston, Texas; Department of Biology, Baylor University, Waco, Texas; James A Baker III Institute of Public Policy, Rice University, Houston, Texas; Scowcroft Institute of International Affairs, Bush School of Government and Public Policy, Texas A&M University, College Station, Texas

Human hookworm infection is one of the most ubiquitous illnesses in people who live in extreme poverty. The Global Burden of Disease (GBD) study estimates that in the year 2016, approximately 450 million people lived with hookworms in their small intestines.^1^ Hookworms feed on human blood and cause long-term intestinal blood loss, leading to iron deficiency.^2^ For individuals with low iron stores, blood-feeding hookworms can cause sufficient iron and blood loss, leading to anemia. A systematic analysis for the GBD study found that a high percentage of anemia disease burden in Oceania and Africa could be attributed to hookworm infection.^3^

Adolescent and adult women are vulnerable to hookworm anemia because of their low iron reserves as a result of menstruation, low iron intake, and other factors.^2^ Pregnant women in resource-poor settings are at added risk of hookworm anemia because of the iron demands of the fetus.^4,5^ Compounding this problem are coinfections from malaria, which together with hookworm infections can result in profound anemia.^6,7^ Indeed, the term “agricultural anemia” was once coined to describe the terrible accumulative effects of hookworms on top of malaria, in the setting of low iron intake and hemoglobinopathies, which are also pervasive in rural Africa.^8^

The adverse consequences of severe hookworm anemia among women in Africa include higher maternal morbidity and mortality.^9^ But another important effect is the impact of hookworm blood loss and anemia on worker productivity. However, this latter effect has been somewhat elusive to measure and assess, even though anecdotally it is often assumed that hookworm anemia is associated with low agricultural productivity.^10,11^ For example, the co-discovery of *Necator americanus* as the etiologic agent of hookworm disease by Bailey K. Ashford, a military physician working in Puerto Rico after the Spanish–American war, was simultaneous with the attribution of hookworms to low worker output.^12,13^ Similar observations were noted by scientists and physicians employed by the Rockefeller Sanitary Commission working in the American South, and in Brazil, China, and elsewhere globally through the Rockefeller International Health Board, which was later named the International Health Division of the Rockefeller Foundation.^14–16^ Indeed, a retrospective analysis of studies conducted in the Southern United States during the early twentieth century found that chronic hookworm infection in children had the ability to stunt not only physical development but also future wage earnings,^17^ presumably because of the effects of chronic hookworm anemia on either on work capacity or intellect or some combination of factors. More recent efforts have looked at hookworms and low productivity among workers harvesting plantation-style crops such as bananas and tea,^18,19^ but the quantitative evidence is still modest and it remains challenging to directly attribute declines in productivity to worms.^20^

To better pin down and measure the associations between worms and work, Baird et al.^21^ looked at school-based deworming programs in Kenya, and combined this analysis with longitudinal data that tracked the school children as they became adults. They confirmed the beneficial effects of deworming for hookworm and other soil-transmitted helminthiases and then proceeded to show that programs of deworming enhanced the education of women and increased their secondary school attendance.^21^ Among men, deworming also increased the labor workforce both qualitatively and quantitatively.^21^ However, the Baird et al. study and related ones have also been criticized for methodological biases,^22^ moreover a Cochrane analysis found that randomized control deworming trials often do not translate to beneficial nutritional and cognitive effects.^23^ Such contradictory findings have led to a vigorous scholarly exchange that is sometimes referred to as “worm wars.”^24,25^

In this issue of the *American Journal of Tropical Medicine and Hygiene*, Salmon et al.^26^ report on a double-blind, prospective randomized effectiveness trial of single-dose albendazole (400 mg) on 250 smallholding women farmers recruited from safe motherhood groups in the Democratic Republic of Congo. Approximately 50% of the women were infected with hookworms, and two-thirds were anemic, including a significant number of women with hemoglobin concentrations less than 10 mg/dL.^26^ The women were randomized into anthelminthic treatment (*N* = 125) and placebo groups (*N* = 125). A major finding was that albendazole treatment of the women was beneficial in terms of aerobic work capacity, even though the effect was not shown to result from increases in blood hemoglobin concentration.^26^

The study is important given the dearth of randomized clinical trials with anthelminthic drugs, especially on adult women and their capacity to work. It also reinforces the importance of considering adult populations in mass drug administration campaigns and extending deworming programs beyond school-aged children to include the entire community.^27,28^ The study here supports the cost-effectiveness rationale of community-wide deworming, as proposed by Anderson and his colleagues,^28^ or a package of interventions that also includes essential medicines for schistosomiasis, lymphatic filariasis, onchocerciasis, trachoma,^29^ and even human immunodeficiency virus/acquired immunodeficiency syndrome and malaria.^30^

In addition, the new study reinforces the removal of hookworms from the human intestine as a potent antipoverty measure. Periodic and frequent deworming using albendazole or mebendazole has been the most widely practiced approach, but immunization against hookworms and other helminth pathogens is also being pursued through the development of so-called antipoverty vaccines.^11,31^ In any case, the Salmon et al. study provides an important piece of evidence base for hookworm prevention as a potent measure to improve the plight of girls and women who live in extreme poverty, and specifically as a means to improve their health and economic well-being. Because of hookworm’s unique effect on agricultural worker productivity in resource-poor economies, hookworm prevention needs to be better prioritized by the world’s finance ministers, and global leaders who wish to introduce or expand interventions that promote women’s health and empowerment.
